# Consolidation Radiotherapy in Stage IE- IIE, Non-Bulky Primary Gastric Diffuse Large B-Cell Lymphoma with Post-Chemotherapy Complete Remission

**DOI:** 10.1371/journal.pone.0133469

**Published:** 2015-07-20

**Authors:** Qiwen Li, Wei Li, Liang Wang, Weida Wang, Shaoqing Niu, Xiwen Bi, Hanyu Wang, Yujing Zhang

**Affiliations:** 1 Department of Radiation Oncology, Sun Yat-sen University Cancer Center, State Key Laboratory of Oncology in South China, Guangzhou, Guangdong, China; 2 Department of Anesthesiology, Sun Yat-sen University Cancer Center, State Key Laboratory of Oncology in South China, Guangzhou, Guangdong, China; 3 Department of Hematologic Oncology, Sun Yat-sen University Cancer Center, State Key Laboratory of Oncology in South China, Guangzhou, Guangdong, China; 4 Department of Radiation Oncology, First Affiliated Hospital of Sun Yat-sen University, Guangzhou, Guangdong, China; 5 Department of Medical Oncology, Sun Yat-sen University Cancer Center, State Key Laboratory of Oncology in South China, Guangzhou, Guangdong, China; Hokkaido University, JAPAN

## Abstract

**Background:**

To investigate the effects of consolidation radiation in patients with stage IE-IIE, non-bulky primary gastric diffuse large B-cell lymphoma (DLBCL).

**Methods:**

A cohort consisted of 71 consecutive patients with stage IE-IIE, non-bulky primary gastric DLBCL was retrospectively analyzed. All of them had been in complete remission after receiving at least four cycles of chemotherapy, containing rituximab or not. Consolidation radiation was delivered thereafter in 28 patients while other 43 received clinical observation only. Locoregional relapse-free survival (LRFS), disease-free survival (DFS), overall survival (OS) and distant metastasis-free survival (DMFS) were compared between patients with or without radiotherapy.

**Results:**

The 10-year LRFS, DFS, OS and DMFS were 100% and 81.4% (*p* = 0.028), 91.7% and 77.1% (*p* = 0.14), 91.7% and 77.8% (*p* = 0.67), 91.7% and 78.0% (*p* = 0.42) for patients with or without radiotherapy.

**Conclusions:**

Radiotherapy is associated with improved locoregional control of patients with early stage primary gastric DLBCL, who have achieved complete remission following at least four cycles of chemotherapy.

## Introduction

30–45% of all extranodal malignant lymphomas is represented by gastric lymphoma [1–2]. Of all gastric lymphomas, primary gastric diffuse large B-cell lymphoma (DLBCL) accounts for about 30%, which is the second most common type of lymphoma occurs in stomach, after mucosa-associated lymphoid tissue lymphoma [3]. The majority of primary gastric DLBCL presents as localized stage, IE/IIE, the optimal therapy of which remains unresolved [4–6].

Treatment modalities for primary gastric DLBCL have altered dramatically during the last decades. Surgery used to play important roles in attaining pathologic specimens, staging and achieving successful local control, but postoperative complications, e.g. dumping syndrome, weight loss, malabsorption syndrome and impairment of quality of life were constantly noticed [7]. Moreover, it is suspected that surgery alone might not be strong enough to prevent systemic recurrence [8]. During recent years, in diagnostic procedures, surgical staging has been replaced by advanced endoscopy and imaging technique. As a treatment, it is no longer obligatory since increasing evidence arose that conservative treatment approach, which has advantage of organ preservation theoretically, leads to comparable or even better clinical outcomes. 10-year overall survival for patients receiving chemotherapy alone reaches to 96%, similar to results of surgery followed by chemotherapy in controlled clinical trials. Local control rate was reported 84–93% in the limited literatures about primary gastric DLBCL [8–10].

As a choice for conservative approaches, radiotherapy (RT) is regarded potentially beneficial in elevating local control and other long-term outcomes. But the most reasonable sequence of multiple treatments is still an open question. Because there does not seem to be any evidence to treat limited-stage primary gastric DLBCL differently from other stage I/II nodal or extranodal DLBCL [11], the principle of treatment for gastric DLBCL follows that of general DLBCLs. Either three cycles of cyclophosphamide, doxorubicin, oncovin, prednisone and rituximab (RCHOP) followed by involved field radiation (IFRT) or long-course chemotherapy (6 cycles of RCHOP) is recommended as standard in National Comprehensive Cancer Network (NCCN) guideline. However, whether RT benefits patients who have achieved complete remission (CR) after at least 4 cycles of chemotherapy remains controversial, especially in the rituximab era. In fact, randomized trial comparing the addition of rituximab or radiation is unlikely to be conducted because of the confirmed survival benefit from immunochemotherapy [12]. Our research aimed at retrospectively investigating the effects of RT in patients with stage IE-IIE, non-bulky primary gastric DLBCL, who have achieved CR after at least four cycles of chemotherapy, including cyclophosphamide, doxorubicin, oncovin, prednisone (CHOP)-like and RCHOP-like regimens.

## Materials and Methods

### Patient characteristics

This study was designed as a retrospective cohort study. Between December 1987 and November 2013, 71 consecutive patients, ≥18 years of age, with Ann Arbor stage IE-IIE pathological confirmed primary gastric DLBCL who were hospitalised at the Sun Yat-sen University Cancer Center were identified. All of them received at least four cycles of chemotherapy and achieved post-chemotherapy CR, followed by involved-field radiotherapy (IFRT) as consolidation. Patients with stage III-IV disease, bulky tumor, history of previous chemotherapy or RT, or history of previous or concurrent malignancy at the time of diagnosis were excluded. The study was reviewed and approved by Ethic Committee of Sun Yat-sen University Cancer Center and conducted according to the principles expressed in the Declaration of Helsinki. Since it was a retrospective analysis of routine data, we requested and were granted a waiver of individual informed consent from Ethic Committee of Sun Yat-sen University Cancer Center.

### Treatments

Chemotherapy used in patients was based on CHOP-like, e.g. CHOP, cyclophosphamide, doxorubicin, oncovin, prednisone, etoposide (ECHOP), or RCHOP-like, e.g. RCHOP, cyclophosphamide, doxorubicin, oncovin, prednisone, etoposide, rituximab (RECHOP) regimens, no less than four cycles. Patients with surgery, no matter radical or palliative, were not excluded. RT was planned as conventional external beam radiation, three-dimensional conformal radiotherapy, or intensity modulated radiotherapy depending on the progress of RT technique in our institute, which started within 3 months after the completion of chemotherapy. The radiated volume included drainage regions with initial positive nodes. The dose of radiation and numbers of chemotherapy cycles were independently decided by patients’ attending oncologists, and retrospectively collected by researchers.

### Response evaluation and follow-up

During chemotherapy, tumor response was evaluated by endoscopy, and contrast enhanced computer tomography or positron emission tomography/computer tomography every two cycles. Clinical complete remission was defined as complete resolution of tumor at endoscopy and on image, which was consistently confirmed by the following examinations. Follow-up evaluation at the outpatient clinic included clinical symptoms assessment, physical examination, laboratory analysis e.g. regular blood test, blood biochemistry test, β_2_-microglobin, and imaging or histological test if necessary, which was performed every 3 months during the first 2 years after treatment, every 6 months in the third year and once a year thereafter.

### Study end points

The primary endpoints of this study were locoregional relapse-free survival (LRFS) and disease-free survival (DFS). The secondary endpoints were overall survival (OS) and distant metastasis-free survival (DMFS). Locoregional recurrence was defined as recurrence occurring in the stomach or regional lymph node drainage area, while other relapse was considered as distant metastasis. DFS was defined as the time from the diagnosis to relapse or death. OS was defined as the time from diagnosis to death from any cause.

### Statistical methods

All endpoints were assessed using Kaplan–Meier method. Univariate analyses were performed by log-rank test. Multivariate analyses were performed using the Cox proportional hazards model. Potential prognostic factors with *p*<0.1 in univariate analyses were included in multivariate analyses. Comparisons of clinico-pathologic variables were performed by Chi-square or Fisher’s exact test for nominal variables. *p*-values <0.05 (two-sided) were considered to be statistically significant. All tests were conducted using SPSS 21.0.

## Results

All data are shown in **S1 Dataset**.

### Patient and treatment characteristics

Clinico-pathologic characteristics based on RT are presented in Table 1. Among 71 patients, 28 received RT and 43 did not. The median RT dose was 36 Gy (range: 21.4–40 Gy). Patients who did not receive RT tended to receive surgery more often (*p* = 0.002). Except for two emergency operations in the case of gastric perforation during chemotherapy, all patients receiving surgery are operated before commence of chemotherapy. Since the early involvement of radical resection, the rate of early CR was higher in patients without RT (*p* = 0.008). The median interval between the end of chemotherapy and start of RT was 43 (range: 15–89) days.There were no differences in the distributions of age, gender, pathological types [13], size of tumor, location, stage, B symptoms, lactate dehydrogenase (LDH), performance status (PS), international prognostic index (IPI), chemotherapy regimens and number of chemotherapy cycles between patients with or without RT.

**Table 1:** Baseline clinico-pathologic characteristics.

Characteristics	RT(n = 28)		Without RT(n = 43)		*p*
	n	%	n	%	
Age					
≥60	6	21.4	10	23.3	*0*.*86*
<60	22	78.6	33	76.7	
Gender					
Male	18	64.3	22	51.2	*0*.*28*
Female	10	35.7	21	48.8	
Pathological type					
GCB	8	42.1	9	56.3	*0*.*51*
Non-GCB	11	57.9	7	43.8	
Missing	9		27		
Tumor size					
≥5 cm	14	66.7	12	36.4	*0*.*3*
<5 cm	7	33.3	21	63.6	
Missing	7		10		
Surgery					
Radical	2	7.1	16	37.2	*0*.*002*
Palliative	1	3.6	6	14	
No surgery	25	89.3	21	48.8	
Location					
Upper	1	3.8	1	2.9	*0*.*86*
Middle	3	11.5	5	14.3	
Lower	12	46.2	19	54.3	
Multiple sites	10	38.5	10	28.6	
Missing	2		8		
Stage					
IE	16	57.1	22	51.2	*0*.*62*
IIE	12	42.9	21	48.8	
B symptom					
Yes	7	25	6	14	*0*.*24*
No	21	75	37	86	
LDH					
High	8	40	4	9.3	*0*.*051*
Normal	20	60	39	90.7	
IPI					
0	14	50	22	51.2	*0*.*92*
1	9	32.1	16	37.2	
2	4	14.3	4	9.3	
3	1	3.6	1	2.3	
PS					
0	21	75	37	86	*0*.*24*
1	7	25	6	14	
CT regimen					
CHOP-like	9	32.1	21	48.8	*0*.*16*
RCHOP-like	19	67.9	22	51.2	
Cycles of CT					
≤4	6	21.4	6	14	*0*.*48*
>4, ≤6	21	75	33	76.7	
>6	1	3.6	4	9.3	
First CR					
After surgery	1	3.6	13	30.2	*0*.*008*
≤2 cycles	6	21.4	10	23.3	
>2, ≤4 cycles	17	60.7	11	25.6	
>4, ≤6 cycles	4	14.3	9	20.9	
RT technique					
Regular	17	60.7			
3D-CRT	8	28.6			
IMRT	3	10.7			
RT dose (Gy)					
≤30	9	32.1			
>30, ≤36	13	46.4			
>36	6	21.4			

GCB = germinal center B; LDH = lactate dehydrogenase; IPI = international prognostic index; PS = Performance Status; CT = chemotherapy; CR = complete remission; RT = radiotherapy; CHOP = cyclophosphamide, doxorubicin, oncovin, prednisone; R = rituximab; 3D-CRT = three-dimensional conformal radiotherapy; IMRT = intensity modulated radiotherapy.

### Failure patterns

The median follow-up duration was 52 (range: 7–265) months for all patients, 50 (range: 7–140) in RT group and 53 (range: 8–265) in non-RT group. During the follow-up, 11(15.4%) patients experienced disease recurrence, 2 (7.1%) in RT group and 9 (20.9%) in non-RT group, respectively. 2 (7.1%) patients in RT group developed distant metastasis, without any cases of locoregional relapse. In non-RT group, locoregional and distal recurrence was found in 7 (16.3%) and 6 (14.0%) patients, respectively. 7 deaths were observed, 2 (7.1%) in RT group and 5 (11.6%) in non-RT group.

### Survival

**Fig 1:**
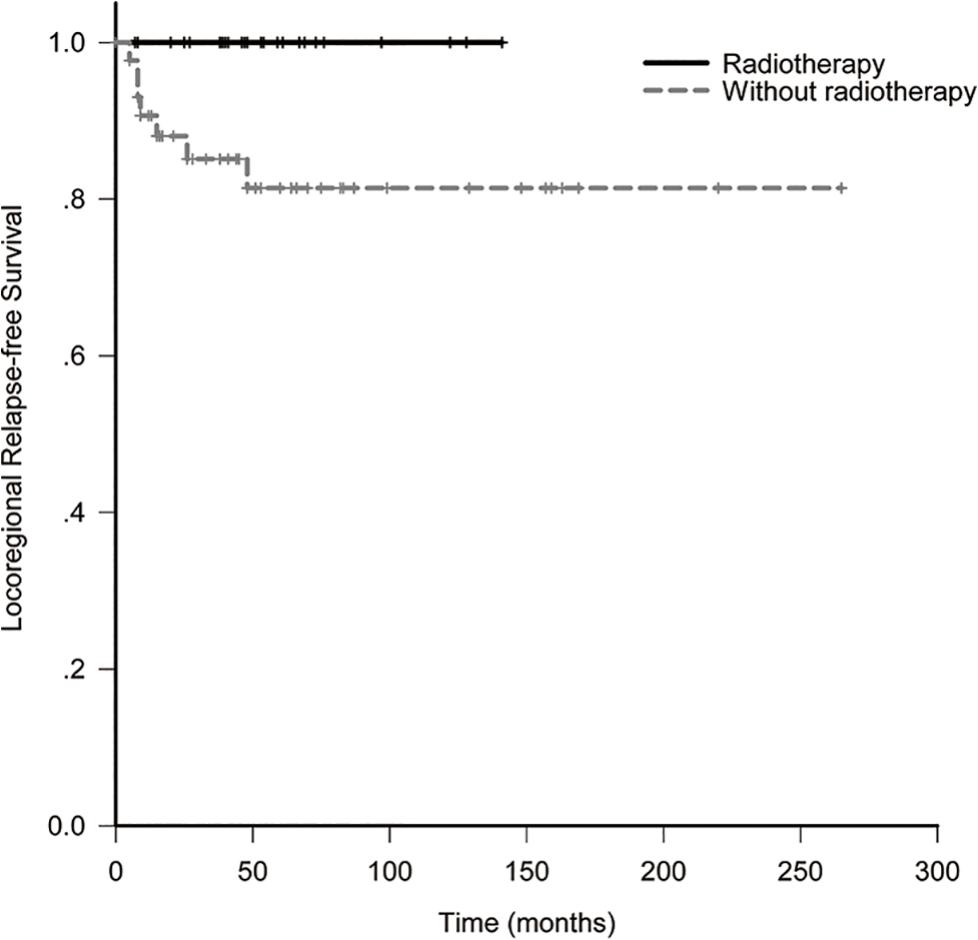
Locoregional relapse-free survival of patients with or without radiotherapy.

**Fig 2:**
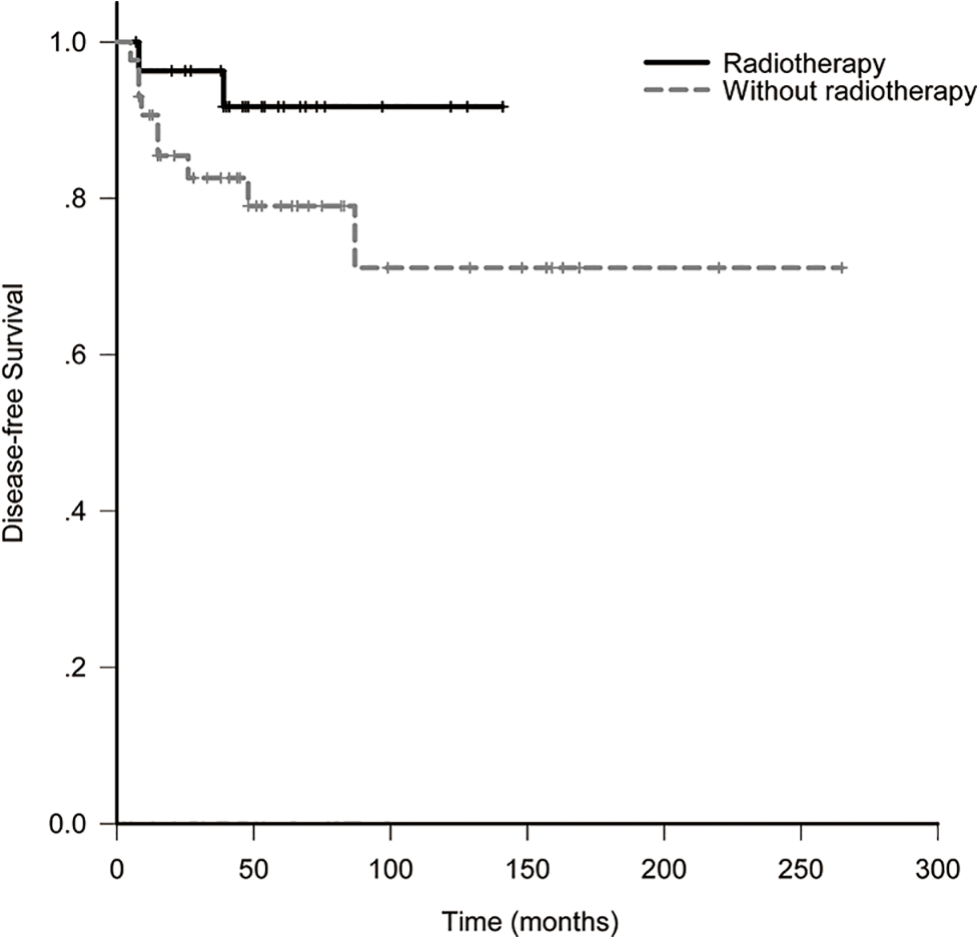
Disease-free survival of patients with or without radiotherapy.

**Fig 3:**
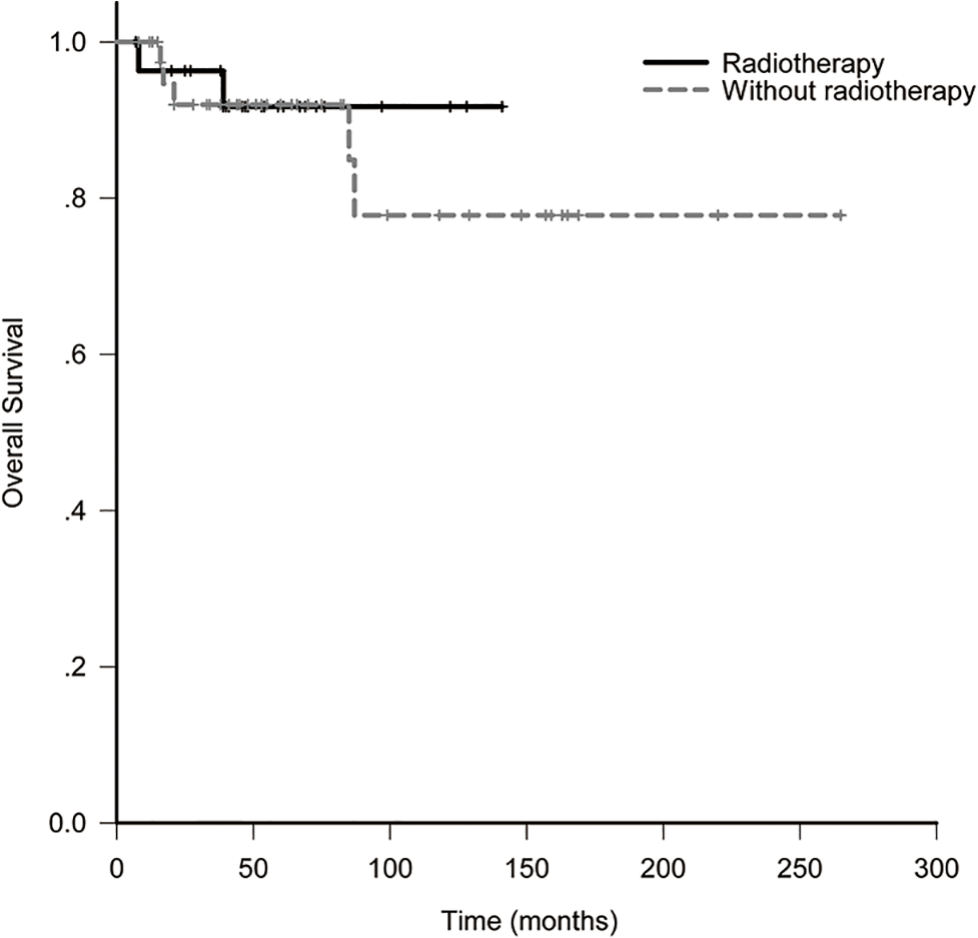
Overall survival of patients with or without radiotherapy.

**Fig 4:**
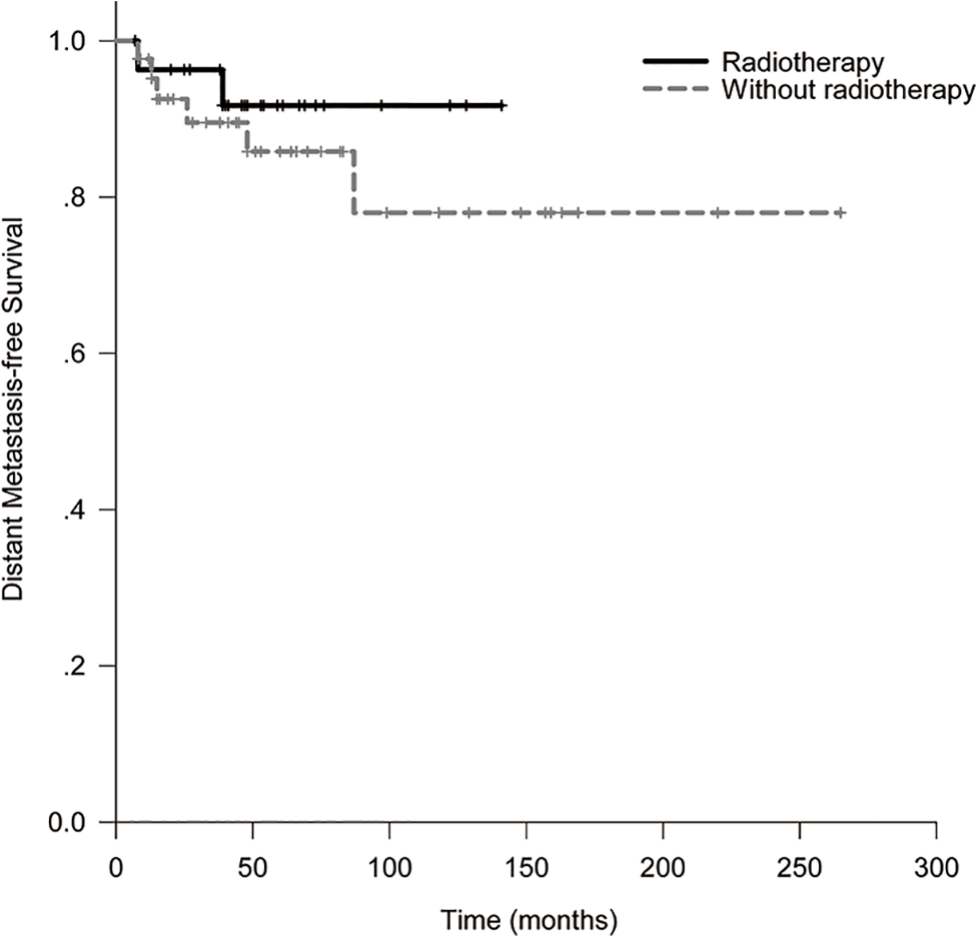
Distant metastasis-free survival of patients with or without radiotherapy.

The 10-year LRFS, DFS, OS and DMFS for all patients were 88.6%, 78.0%, 81.2% and 82.3%. In RT and non-RT group, they were 100% and 81.4% (*p* = 0.028, Fig 1), 91.7% and 77.1% (*p* = 0.14, Fig 2), 91.7% and 77.8% (*p* = 0.67, Fig 3), 91.7% and 78.0% (*p* = 0.42, Fig 4). When analyzed by univariate, DFS was affected by stage (5-year DFS: IE *vs* IIE: 94.1% *vs* 60.4%, *p* = 0.012) and IPI (5-year DFS: 0 *vs* 1 *vs* 2 *vs* 3: 84.6% *vs* 86.6% *vs* 87.5% *vs* 0%, *p* = 0.017). DMFS was affected by IPI (5-year DMFS: 0 *vs* 1 *vs* 2 *vs* 3: 90.5% *vs* 90.2% *vs* 87.5% *vs* 0%, *p* = 0.002). OS was affected by stage (5-year OS: IE vs. IIE: 100% *vs* 62.3%, *p* = 0.004) and IPI (5-year OS: 0 *vs* 1 *vs* 2 *vs* 3: 96.8% *vs* 73.0% *vs* 87.5% *vs* 0%, *p* = 0.001). None of other clinico-pathologic factors, except for radiotherapy, was statistically related to LRFS (5-year LRFS: RT *vs* non-RT: 100% *vs*. 81.4%, *p* = 0.028, Table 2). On multivariate analysis, stage was identified as an independent prognostic factors for DFS, with hazard ratio 0.18 (95% confidence interval: 0.036–0.897, *p* = 0.036). Unfortunately, the numbers of events of interest for LRFS, OS and DMFS are not enough to allow multivariate analysis in the study.

**Table 2:** Univariate analysis of prognostic factors.

Variable	n	5-y LRFS (%)	*p*	5-y DFS (%)	*p*	5-y OS (%)	*p*	5-y DMFS (%)	*p*
All patients	71	88.6		78		81.2		82.3	
Age									
≥60	16	84.4	*0*.*74*	77.9	*0*.*67*	86.2	*0*.*1*	77.9	*0*.*28*
<60	55	90		86.5		94.1		92	
Gender									
Male	40	90.2	*0*.*39*	84.8	*0*.*73*	94.1	*0*.*87*	84.8	*0*.*35*
Female	31	86.1		83		89.2		93.1	
Pathological type									
GCB	17	91.7	*0*.*9*	91.7	*0*.*66*	100	*0*.*35*	91.7	*0*.*9*
Non-GCB	18	94.1		88.9		94.4		94.4	
Tumor size									
≥5 cm	19	87.2	*0*.*62*	82.6	*0*.*61*	89.2	*0*.*51*	87.4	*0*.*95*
<5 cm	35	94.2		87		89.3		86.8	
Surgery									
Radical	18	81.5	*0*.*51*	76	*0*.*49*	87.8	*0*.*29*	86.5	*0*.*46*
Palliative	7	83.3		83.3		100		83.3	
No surgery	46	93.2		88.1		92.4		90.2	
Location									
Upper	2	50	*0*.*07*	50	*0*.*53*	50	*0*.*35*	50	*0*.*31*
Middle	8	75		75		100		100	
Lower	31	86.1		82.7		96		82.7	
Multiple sites	20	94.7		84.4		84.1		89.1	
Stage									
IE	38	94.1	*0*.*15*	94.1	*0*.*012*	100	*0*.*004*	94.1	*0*.*09*
IIE	33	82		60.4		83.1		81.2	
B symptom									
Yes	13	100	*0*.*22*	91.7	*0*.*5*	91.7	*0*.*91*	91.7	*0*.*8*
No	58	86.3		82.2		91.9		87.2	
LDH									
High	12	90.9	*0*.*86*	74.1	*0*.*42*	73.3	*0*.*11*	81.5	*0*.*6*
Normal	59	88.1		86.3		96.2		89.8	
IPI									
0	36	87.6	*0*.*75*	84.6	*0*.*017*	96.8	*0*.*001*	90.5	*0*.*002*
1	25	86.6		86.6		91.3		90.2	
2	8	100		87.5		87.5		87.5	
3	2	100		0		50		0	
PS									
0	58	87.4	*0*.*7*	81.7	*0*.*35*	90	*0*.*16*	86.7	*0*.*58*
1	13	92.3		92.3		100		92.3	
CT regimen									
CHOP-like	30	80.5	*0*.*12*	73.4	*0*.*068*	88.3	*0*.*37*	79.6	*0*.*11*
RCHOP-like	41	94.8		92.3		94.8		94.9	
Cycles of CT									
4	12	83.3	*0*.*55*	83.3	*0*.*64*	81.8	*0*.*42*	90.9	*0*.*72*
>4, ≤6	54	88.8		82.9		93.7		86.6	
>6	5	100		100		100		100	
First CR									
After surgery	14	68.6	*0*.*11*	68.6	*0*.*56*	92.3	*0*.*93*	81.8	*0*.*75*
≤2 cycles	16	93.8		93.8		92.9		93.3	
>2, ≤4 cycles	28	96.3		83.4		87		83.4	
>4, ≤6 cycles	13	91.7		91.7		100		100	
RT									
Yes	28	100	*0*.*028*	91.7	*0*.*14*	91.7	*0*.*67*	91.7	*0*.*42*
No	43	81.4		79		92		85.8	
RT technique									
Regular	17	100	*1*	87.1	*0*.*55*	87.1	*0*.*55*	87.1	*0*.*55*
3D-CRT	8	100		100		100		100	
IMRT	3	100		100		100		100	
RT dose (Gy)									
≤30	9	100	*1*	100	*0*.*3*	100	*0*.*3*	100	*0*.*3*
>30, ≤36	13	100		82.1		82.1		82.1	
>36	6	100		100		100		100	

GCB = germinal center B; LDH = lactate dehydrogenase; IPI = international prognostic sindex; PS = Performance Status; CT = chemotherapy; CR = complete remission, RT = radiotherapy; CHOP = cyclophosphamide, doxorubicin, oncovin, prednisone; R = rituximab.LRFS = locoregional relapse-free survival; DFS = disease-free survival; OS = overall survival; DMFS = distant metastasis-free survival; 3D-CRT = three-dimensional conformal radiotherapy; IMRT = intensity modulated radiotherapy

## Discussion

The optimal treatment for stage IE-IIE primary gastric DLBCL is under constant debate. As a subtype of DLBCL, it has become evident that either short-course chemotherapy plus IFRT or full-course chemotherapy alone are appropriate options. In Southwest Oncology Group (SWOG) 8736 study, compared with eight courses of CHOP, combining three courses of CHOP with IFRT revealed OS benefit at 5 years, which lasted for almost 10 years before losing statistical significance in older patients. It is reported that patients with no risk factors had satisfactory 5-year OS rates, approximately 94% when treated with chemotherapy+RT [14]. In another Canadian study, 5-year survival was reported 95% in patients under 69 years old and without any risk factors, treated with CHOP and IFRT [15]. However, considering the effect of consolidation after long-course chemotherapy, there were two controversial clinical trials. The Eastern Cooperative Oncology Group (ECOG) study 1484 attempted to solve out whether IFRT as consolidation after eight cycles of CHOP led to any benefit. In analysis of recurrence patterns, the study demonstrated a marginal benefit of local control in CR patients receiving IFRT (*p* = 0.06). 6-year disease-free survival was significantly elevated in the IFRT group (70% vs 56%; *p* = 0.05). But this study was widely questioned regarding its high drop-out rate and absence of the information of causes of death [16]. Another research, the Groupe d’Etude des Lymphomes de l’Adulte (GELA) LNH 93–4 study was designed to answer if consolidation RT was superior to observation after four cycles of CHOP in old patients with stage IE-IIE aggressive DLBCL [17]. Similarly, it suggested a definite improvement in local control from the addition of RT, revealing relapsing rate in patients with local failure 63% after CHOP alone, versus 34% after CHOP plus consolidation RT, although no advantage of RT was identified on event-free survival (5-year event-free survival: 64% *vs* 61%, *p* = 0.06) or OS (5-year OS: 68% *vs* 65%, *p* = 0.24). However, the conclusion was quite limited due to poor adherence of protocol. In fact, 40% of participants were under-dosed or had an inappropriate delay of 3 months between chemotherapy and RT, 12% of whom even failed to receive any radiation. Moreover, the patient group receiving consolidation RT had a remarkably inferior outcome compared to those treated in ECOG study 1484, inferring weakness in study management. Another uncertainty is, with the introduction of immunochemotherapy, previous evidence was questioned because none of them include the use of rituximab due to early conduct. Such limitations make the benefit of consolidation RT after at least four cycles’ chemotherapy in the rituximab era remains unsolved.

Following systemic therapy alone in patients with DLBCL, the most common pattern of initial failure is local recurrence, compromising 41–63% of all relapses [16–18]. Focusing on localized DLBCL of the stomach, 5-year overall survival reported in literatures ranges from 79% to 93% [19–22]. Our study revealed 10-year DFS and OS 78.0% and 81.2%, which is not worse than results from previous studies. Though clinical benefits from RT on DFS and OS were not statistically significant, a favored LRFS was concluded (100% and 81.4%, *p* = 0.028). These results fit perfectly with a randomized controlled trial published by Martinelli et al, in which 45 patients with primary gastric DLBCL were randomly assigned to RT or another 2 cycles of CHOP when they achieved CR after 4–6 cycles of CHOP. Likewise, it demonstrated consolidation RT significantly reduced the risk of local relapse from 13% to 0% and improved DFS, while OS was similar between groups [10].

Chemotherapy combined with immunotherapy has evolved to be the backbone of treatment for systemic disease control. MinT trial revealed, in patients with non-bulky disease and IPI of 0, 6-year event-free survival, progression-free survival and overall survival after chemotherapy plus rituximab was 84.3%, 89.6%, and 94.9%, respectively [12]. Comparing results from SWOG 0014 to historical controls in the SWOG 8736 study, rituximab seemed to improve two-year progression-free survival beyond that achieved by short-course chemotherapy and consolidation RT alone in patients with limited stage DLBCL [23]. Some have speculated that rituximab may eventually take place of IFRT in the management of early stage DLBCL. Biologically, in the treatment of lymphoma, it lacks evidence that targeting CD20 can actually take the place of the effects of ionizing radiation. In our research, more than half (58%) of participants were prescribed RCHOP-like regimens, which distributed statistically balanced between RT and non-RT groups. Although subgroup analysis was hard to perform because of limited sample, it inferred that benefit of RT on local control may still exist when rituximab was adopted. Likewise, Phan et al studied 469 patients with staged I-IV DLBCL and received at least six cycles of R-CHOP. They concluded radiotherapy remained to be a significant favorable factor influencing OS and PFS in multivariate analyses [24]. Thus, the role of RT in rituximab era is potentially beneficial, which is to be further studied in well-designed controlled trials.

It is difficult to get full access to information of toxic effects in our retrospective data. However, in Martinellis’ report, when 30 Gy was delivered to the stomach and involved drainage regions, no severe complications, including renal toxicity, were noticed. Only a few patients were disturbed with mild nausea [10].

In our research, most important known prognostic factors were well balanced between groups. However, in non-RT patients, surgery was more likely to be involved. As a result, pre-chemotherapy CR was achieved in more than 30% of non-RT patients, which was significantly higher than that of RT patients. Such difference was not considered as important confounding factors because according to early studies, based on sufficient chemotherapy, whether surgery was performed did not seem to affect event-free survival or OS, neither the pattern of recurrence [8]. Besides, although non-RT group was related to higher rate of surgery and tended to have early CR, RT group still turned out to have favored local control, furtherly implying the advantage of consolidation RT.

## Conclusions

To summarize, in this retrospective cohort study, we show for the first time that radiotherapy is associated with improved locoregional control of patients with early stage primary gastric DLBCL, after chemotherapy of ≥four cycles, even when rituximab was adopted.

## Supporting Information

S1 Dataset Research data. (XLSX)

## References

[1] d'Amore F, Brincker H, Grønbaek K, Thorling K, Pedersen M, Jensen MK, et al Non-Hodgkin's lymphoma of the gastrointestinal tract: a population-based analysis of incidence, geographic distribution, clinicopathologic presentation features, and prognosis. Danish Lymphoma Study Group. J Clin Oncol. 1994; 12:1673–1684. 8040680 10.1200/JCO.1994.12.8.1673

[2] Herrmann R, Panahon AM, Barcos MP, Walsh D, Stutzman L. Gastrointestinal involvement in non-Hodgkin's lymphoma. Cancer. 1980;46:215–222. 7388763 10.1002/1097-0142(19800701)46:1<215::aid-cncr2820460136>3.0.co;2-6

[3] Mihaljević B, Nedeljkov-Jancić R, Vujicić V, Antić D, Janković S, Colović N. Primary extranodal lymphomas of gastrointestinal localizations: a single institution 5-yr experience. Med Oncol. 2006;23:225–235. 16720923 10.1385/MO:23:2:225

[4] Koch P, del Valle F, Berdel WE, Willich NA, Reers B, Hiddemann W, et al Primary gastrointestinal non-Hodgkin's lymphoma: I. Anatomic and histologic distribution, clinical features, and survival data of 371 patients registered in the German Multicenter Study GIT NHL 01/92. J Clin Oncol. 2001;19:3861–3873. 11559724 10.1200/JCO.2001.19.18.3861

[5] Brooks JJ, Enterline HT. Primary gastric lymphomas. A clinicopathologic study of 58 cases with long-term follow-up and literature review. Cancer. 1983;51:701–711. 6336982 10.1002/1097-0142(19830215)51:4<701::aid-cncr2820510425>3.0.co;2-d

[6] Amer MH, el-Akkad S. Gastrointestinal lymphoma in adults: clinical features and management of 300 cases. Gastroenterology. 1994;106:846–858. 8143991 10.1016/0016-5085(94)90742-0

[7] Fischbach W, Schramm S, Goebeler E. Outcome and quality of life favour a conservative treatment of patients with primary gastriclymphoma. Z Gastroenterol. 2011;49:430–435. 10.1055/s-0029-1246012 21476178

[8] Avilés A, Nambo MJ, Neri N, Huerta-Guzmán J, Cuadra I, Alvarado I, et al The role of surgery in primary gastric lymphoma: results of a controlled clinical trial. Ann Surg. 2004;240:44–50. 15213617 10.1097/01.sla.0000129354.31318.f1 PMC1356373

[9] Ferreri AJ, Cordio S, Ponzoni M, Villa E. Non-surgical treatment with primary chemotherapy, with or without radiation therapy, of stage I-II high-grade gastric lymphoma. Leuk Lymphoma. 1999;33:531–541. 10342580 10.3109/10428199909058457

[10] Martinelli G, Gigli F, Calabrese L, Ferrucci PF, Zucca E, Crosta C, et al Early stage gastric diffuse large B-cell lymphomas: results of a randomized trial comparing chemotherapy alone versus chemotherapy + involved field radiotherapy. Leuk Lymphoma. 2009;50:925–931. 10.1080/10428190902912478 19479614

[11] Narita M, Yatabe Y, Asai J, Mori N. Primary gastric lymphomas: morphologic, immunohistochemical and immunogenetic analyses. Pathol Int 1996;46:623–629. 8905870 10.1111/j.1440-1827.1996.tb03664.x

[12] Pfreundschuh M, Kuhnt E, Trümper L, Osterborg A, Trneny M, Shepherd L, et al MabThera International Trial (MInT) Group. CHOP-like chemotherapy with or without rituximab in young patients with good-prognosis diffuse large-B-cell lymphoma: 6-year results of an open-label randomised study of the MabThera International Trial (MInT) Group. Lancet Oncol. 2011;12:1013–1022. 10.1016/S1470-2045(11)70235-2 21940214

[13] Hans CP, Weisenburger DD, Greiner TC, Gascoyne RD, Delabie J, Ott G, et al Confirmation of the molecular classification of diffuse large B-cell lymphoma by immunohistochemistry using a tissue microarray. Blood. 2004;103:275–282. 14504078 10.1182/blood-2003-05-1545

[14] Miller TP, Dahlberg S, Cassady JR, Adelstein DJ, Spier CM, Grogan TM, et al Chemotherapy alone compared with chemotherapy plus radiotherapy for localized intermediate- and high-grade non-Hodgkin's lymphoma. N Engl J Med. 1998;339:21–26. 9647875 10.1056/NEJM199807023390104

[15] Shenkier TN, Voss N, Fairey R, Gascoyne RD, Hoskins P, Klasa R, et al Brief chemotherapy and involved-region irradiation for limited-stage diffuse large-cell lymphoma: an 18-year experience from the British Columbia Cancer Agency. J Clin Oncol. 2002;20:197–204. 11773170 10.1200/JCO.2002.20.1.197

[16] Horning SJ, Weller E, Kim K, Earle JD, O'Connell MJ, Habermann TM, et al Chemotherapy with or without radiotherapy in limited-stage diffuse aggressive non-Hodgkin's lymphoma: Eastern Cooperative Oncology Group study 1484. J Clin Oncol. 2004;22:3032–3038. 15210738 10.1200/JCO.2004.06.088

[17] Bonnet C, Fillet G, Mounier N, Ganem G, Molina TJ, Thiéblemont C, et al Groupe d'Etude des Lymphomes de l'Adulte. CHOP alone compared with CHOP plus radiotherapy for localized aggressive lymphoma in elderly patients: a study by the Groupe d'Etude des Lymphomes de l'Adulte. J Clin Oncol. 2007;25:787–792. 17228021 10.1200/JCO.2006.07.0722

[18] Reyes F, Lepage E, Ganem G, Molina TJ, Brice P, Coiffier B, et al ACVBP versus CHOP plus radiotherapy for localized aggressive lymphoma. N Engl J Med. 2005;352:1197–1205. 15788496 10.1056/NEJMoa042040

[19] Liu H, Zhang RP, Li FX, Quan JC, Liang H. Treatment and prognostic analysis of early stage of primary gastric diffuse large B-cell lymphoma. Zhonghua Wei Chang Wai Ke Za Zhi. 2013;16:36–39. 23355237

[20] Sbitti Y, Ismaili N, Bensouda Y, Kadiri H, Ichou M, Errihani H. Management of stage one and two-E gastric large B-cell lymphoma: chemotherapy alone or surgery followed by chemotherapy? J Hematol Oncol. 2010;3:23 10.1186/1756-8722-3-23 20569496 PMC2901218

[21] Mafune KI, Tanaka Y, Suda Y, Izumo T. Outcome of patients with non-Hodgkin's lymphoma of the stomach after gastrectomy: clinicopathologic study and reclassification according to the revised European-American lymphomaclassification. Gastric Cancer. 2001;4:137–143. 11760079 10.1007/pl00011736

[22] Liu HT, Hsu C, Chen CL, Chiang IP, Chen LT, Chen YC. Chemotherapy alone versus surgery followed by chemotherapy for stage I/IIE large-cell lymphoma of the stomach. Am J Hematol. 2000;64:175–179. 10861812 10.1002/1096-8652(200007)64:3<175::aid-ajh6>3.0.co;2-7

[23] Persky DO, Unger JM, Spier CM, Stea B, LeBlanc M, McCarty MJ, et al Phase II study of rituximab plus three cycles of CHOP and involved-field radiotherapy for patients withlimited-stage aggressive B-cell lymphoma: Southwest Oncology Group study 0014. J Clin Oncol. 2008;26:2258–2263. 10.1200/JCO.2007.13.6929 18413640

[24] Phan J, Mazloom A, Medeiros LJ, Zreik TG, Wogan C, Shihadeh F, et al Benefit of consolidative radiation therapy in patients with diffuse large B-cell lymphoma treated with R-CHOP chemotherapy. J Clin Oncol. 2010;28:4170–4176. 10.1200/JCO.2009.27.3441 20713859

